# Undulation of a moving fluid membrane pushed by filament growth

**DOI:** 10.1038/s41598-021-87073-6

**Published:** 2021-04-12

**Authors:** Hiroshi Noguchi, Olivier Pierre-Louis

**Affiliations:** 1 Institute for Solid State Physics, University of Tokyo, Kashiwa Chiba, 277-8581 Japan; 2 Institut Lumière Matière, UMR5306 Université Lyon 1-CNRS, Université de Lyon, 69622 Villeurbanne, France

**Keywords:** Biological physics, Statistical physics, thermodynamics and nonlinear dynamics, Membrane biophysics

## Abstract

Biomembranes experience out-of-equilibrium conditions in living cells. Their undulation spectra are different from those in thermal equilibrium. Here, we report on the undulation of a fluid membrane pushed by the stepwise growth of filaments as in the leading edge of migrating cells, using three-dimensional Monte Carlo simulations. The undulations are largely modified from equilibrium behavior. When the tension is constrained, the low-wave-number modes are suppressed or enhanced at small or large growth step sizes, respectively, for high membrane surface tensions. In contrast, they are always suppressed for the tensionless membrane, wherein the wave-number range of the suppression depends on the step size. When the membrane area is constrained, in addition to these features, a specific mode is excited for zero and low surface tensions. The reduction of the undulation first induces membrane buckling at the lowest wave-number, and subsequently, other modes are excited, leading to a steady state.

In nonequilibrium, surfaces and interfaces often exhibit different fluctuations from thermal equilibrium. The fluctuations of growing surfaces have been studied by the Kardar–Parisi–Zhang equation and other models^1–7^. Scaling laws for the spatial and temporal evolution of surface roughness and correlation functions have been intensively discussed in various physical systems. On the other hand, larger undulations of fluid membranes have been observed out of equilibrium than in equilibrium^8–11^. For example, red blood cells exhibit non-thermal fluctuations^10^. However, nonequilibrium membrane undulations have only been investigated under limited conditions.

In living cells, membranes often interact with protein filaments (e.g., actin^12,13^ and microtubules^14,15^), which push and/or pull the membranes. For example, actin filaments grow at the front side of crawling cells and push the membrane forward. This membrane motion has been intensively studied by Brownian ratchet theories and simulations^15–28^. However, membrane fluctuations have not yet been studied. In most studies reported in the literature^16–24^, the membrane is modeled as a flat rigid surface; thus, no fluctuations are accounted for. Although the membrane fluctuations are assumed to be a Gaussian distribution of the membrane height in Ref.^25^, the spatial correlation was not accounted for. Membrane fluctuations were also considered with a one-dimensional lattice model^26–28^, but the surface tension was treated by a rough approximation of the rectangular contour length in the absence of the bending energy. Thus, the effects of the bending rigidity have not yet been investigated.

In this study, we report on a minimal model that describes the fluctuations of a fluid membrane with the bending rigidity in interaction with growing filaments. We examine the fluctuations of the membrane pushed by filament growth using Monte Carlo (MC) simulations. The filaments grow by a stepwise random walk under the membrane and have an excluded-volume interaction with the membrane. The analysis of the fluctuation spectrum reveals that membranes deviate from the well-known equilibrium behavior under either condition in which the bending energy or surface tension is dominant. Fluctuations are enhanced or suppressed depending on the conditions. Moreover, enhancement of a specific wave-number is obtained when the membrane area is constrained.

## Results

**Figure 1:**
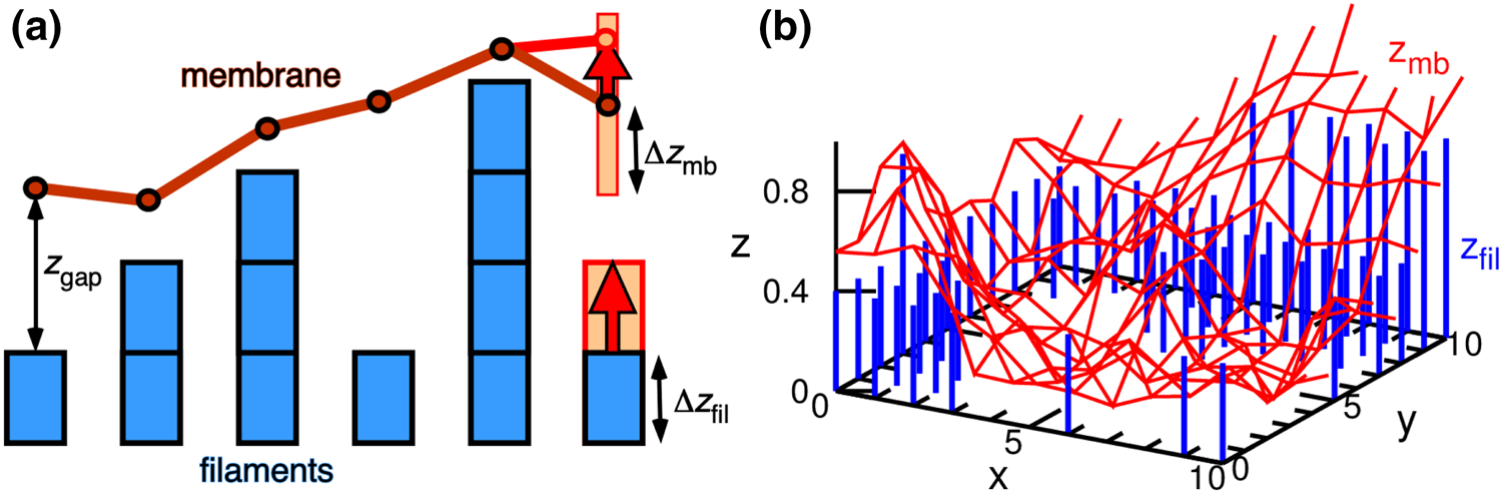
Motion of the membrane and filaments. (**a**) Schematic of the membrane and filaments. According to the Metropolis MC method, the membrane moves with a vertical (*z*) step taken from a uniform random number in $[-\Delta z_{\mathrm{mb}},\Delta z_{\mathrm{mb}}]$. The filament grows stepwise with a probability $p_{\mathrm{fil}}$. (**b**) Snapshot of a part of the membrane and filaments at ${\Delta z_{{{\text{fil}}}}} = 0.4$, $p_{\mathrm{fil}}=0.5$, $\gamma =0$, and $N=4096$ in case I (tension constraint). The red mesh and blue bars represent the membrane and filaments, respectively. The origin of the *z* coordinate is taken at the minimum value of the filament position $z_{\mathrm{fil}}$.

The filaments are modeled as a square array of columns growing vertically in the *z* direction by the addition of new growth units of $\Delta z_{\mathrm{fil}}$. The filaments grow only upward in the $+z$ direction with a probability $p_{\mathrm{fil}}$, as shown in Fig. 1. The filament retraction and interactions between filaments are not considered for simplicity. The fluid membrane is described by a continuous height *z*, defined on a square mesh above each filament, and is moved stochastically upward and downward by the Metropolis MC method. Unless otherwise specified, the number $N=64^2=4096$ of filaments (as well as membrane vertices) and the filament growth probability $p_{\mathrm{fil}}=0.5$ are used (see “Methods” for more details). The results are displayed with the thermal energy $k_{\mathrm{B}}T$ and lateral distance between neighboring filaments $\ell _{\mathrm{fil}}$ for the energy and length units, respectively.

### Thermal equilibrium

**Figure 2:**
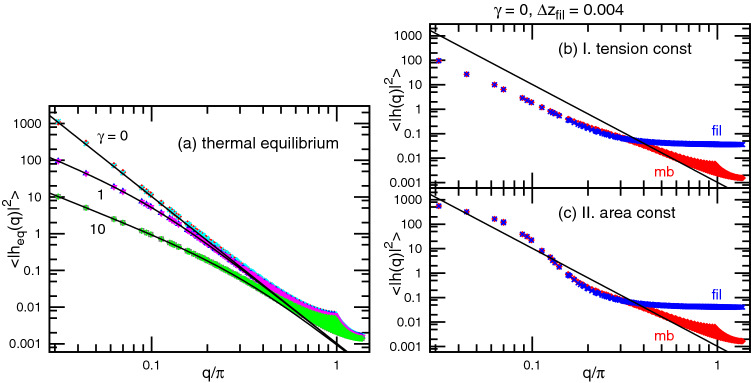
Spectra of undulation modes $\langle |h(q)|^2 \rangle$. (**a**) Membrane under thermal equilibrium at $\gamma =0$, 1, and 10. The symbols ($\times$, $\triangledown$, and $+$) and ($\diamond$, $\triangle$, and $\Box$) represent the spectra in cases I (tension constraint) and II (area constraint), respectively. Solid lines show $1/(\kappa q^4+\gamma q^2)$ with $\kappa =10$. (**b**, **c**) The undulation spectra of the membrane (red diamonds) and filament tips (blue cross marks) of tensionless membranes ($\gamma =0$) at $\Delta z_{\mathrm{fil}}=0.004$ for (**b**) case I and (**c**) case II. The error bars are smaller than the symbol size.

In thermal equilibrium (no interactions with the filaments), the height $z=h(x,y)$ of the membrane exhibits a static spectrum that is controlled by the bending energy and tension^29–32^:

1 $$\begin{aligned} \langle |h(q)|^2 \rangle = \frac{1}{\kappa q^4 + \gamma q^2}, \end{aligned}$$

where *q* is the norm of the wave-vector in the *x*, *y* plane, $\kappa$ is the bending rigidity, and $\gamma$ is the mechanical surface tension^33^ conjugated to the projected membrane area $A_{xy}=N$, respectively (see Fig. 2a). For low wave-numbers ($q \lesssim \sqrt{\gamma /\kappa }$), the tension is the dominant factor in determining membrane undulation, while the bending rigidity is at high wave-numbers ($q \gtrsim \sqrt{\gamma /\kappa }$). In this study, $\kappa = 10$ is used. Thus, for $\gamma =10$, surface tension is dominant for most of the wave-number range; for $\gamma =1$, the tension and bending energies are dominant for low and high wave-numbers, respectively. To maintain the surface tension or membrane area, we employ two types of constraints: case I (tension constraint), in which the intrinsic surface tension $\gamma _{\mathrm{it}}$ conjugated to the real membrane area *A* is imposed by the potential $U_{\mathrm{it}} =\gamma _{\mathrm{it}}A$, and case II (area constraint), in which area *A* is constrained by a harmonic potential $U_{\mathrm{ar}}=(K_A/2A_0)(A-A_0)^2$, where $K_A$ is the area compression modulus. In these cases, ${\gamma_{\mathrm{it}}}$ or $A_0$ is adjusted to obtain the spectrum given by Eq. (1). Note that $\gamma _{\mathrm{it}}$ is slightly greater than $\gamma$ because of the membrane undulation as discussed in the literature^33–36^. In case I, the membrane is a part of a cell membrane or liposome and the rest part of the membrane can act as a membrane reservoir to change the area, while no reservoir exist in case II. Both types of constraints lead to the same static spectrum as shown in Fig. 2a.

### Steady-state velocity

**Figure 3:**
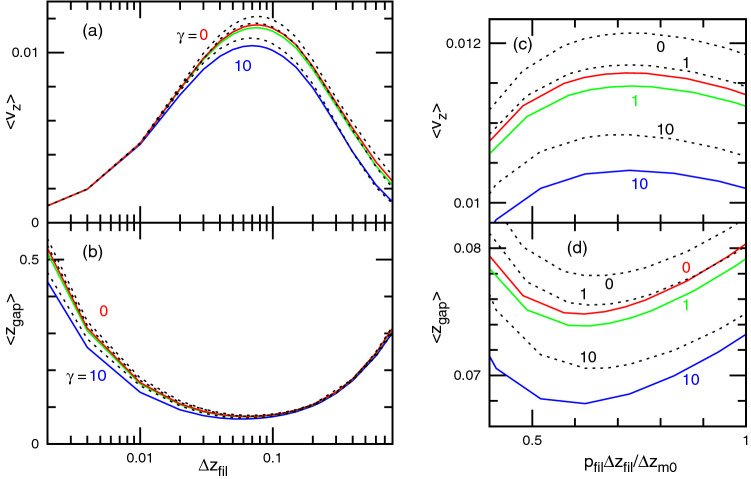
Mean growth velocity $\langle v_z\rangle$ and mean distance between the membrane and filament tips $\langle z_{\mathrm{gap}} \rangle$ . In (a) and (b), these quantities are plotted as a function of $\Delta z_{\mathrm{fil}}$ for $\gamma =0$, 1, and 10. The solid and dashed lines represent the data in cases I (tension constraint) and II (area constraint), respectively. In (**c**) and (**d**), the data around the maximum velocity and minimum distance are enlarged, and the mean filament growth distance $p_{{{\text{fil}}}} {\Delta z_{{{\text{fil}}}}}$ normalized by the mean distance $\Delta z_{\mathrm{m0}}$ of the membrane motion at one MC step. The error bars are smaller than the line thickness.

As the filaments push the membrane upward, the membrane and filament tips relax to a steady-state, in which they move at the same speed $\langle v_z\rangle$. The growth speed $\langle v_z\rangle$ exhibits a non-monotonic dependence on the size of the filament growth units $\Delta z_{\mathrm{fil}}$. For intermediate values of ${\Delta z_{\mathrm{fil}}}$, a maximum speed is reached concomitantly with a minimum in the average of the gap distance $z_{\mathrm{gap}}=z_{\mathrm{mb}}-z_{\mathrm{fil}}$ between the membrane and filament tips, as shown in Fig. 3. Hence, the averaged motion is not sensitive to the surface tension $\gamma$ or to the constraint types (case I or II). In all cases a similar dependence on $\Delta z_{\mathrm{fil}}$ is found.

The two asymptotic regimes of large and small $\Delta z_{\mathrm{fil}}$ present distinct behaviors. For a large step of $\Delta z_{\mathrm{fil}}$, the mean growth velocity $\langle v_z\rangle$ and the gap distance $\langle z_{\mathrm{gap}} \rangle$ are independent of $p_{\mathrm{fil}}$ and determined by $\Delta z_{\mathrm{fil}}$, as shown in Fig. S1, because frequent growth trials of the filaments are rejected until the membrane moves by a sufficiently large distance for the filament growth units to be inserted. This corresponds to the diffusion limit in the Brownian ratchet model^16,17^. On the other hand, the behavior in response to small steps of $\Delta z_{\mathrm{fil}}$ is predominantly determined by the ratio of the filament growth rate $p_{\mathrm{fil}}\Delta z_{\mathrm{fil}}$ to the mean membrane step size $\Delta z_{\mathrm{m0}}=p_{\mathrm{mb}}\Delta z_{\mathrm{mb}}/2$, where $p_{\mathrm{mb}}$ is the mean acceptance ratio of the membrane motion in the absence of filaments ($p_{\mathrm{mb}}\approx 0.5$ in our simulations, see “Methods”). The velocity $\langle v_z\rangle$ for $p_{\mathrm{fil}}=0.25$, 0.5, and 1 merge into one curve when they are plotted with the horizontal axis of $p_{\mathrm{fil}}\Delta z_{\mathrm{fil}}/\Delta z_{\mathrm{m0}}$ (see Fig. S1d), whereas a small difference remains in $\langle z_{\mathrm{gap}} \rangle$ (see Fig. S1e). The velocity approaches the growth velocity of free filaments, $p_{\mathrm{fil}}\Delta z_{\mathrm{fil}}$, for $\Delta z_{\mathrm{fil}}\rightarrow 0$, so that this corresponds to the reaction limit in the Brownian ratchet model^16,17^. The maximum velocity and minimum distance are obtained at $p_{\mathrm{fil}}\Delta z_{\mathrm{fil}} \sim \Delta z_{\mathrm{m0}}$, i.e., when the motion of the membrane opens gaps corresponding to the size of filament growth units at a frequency which is similar to that of the insertion of new growth units (see Fig. 3c and d as well as Fig. S1d and e). Furthermore, irrespective of the average value of the gap, the probability distribution $P(z_{\mathrm{gap}})$ of the gap $z_{\mathrm{gap}}$ decreases monotonically with increasing $z_{\mathrm{gap}}$ and exhibits a stepwise discontinuity at $z_{\mathrm{gap}}=\Delta z_{\mathrm{fil}}$, because the filament growth is rejected at a smaller distance (see Fig. S2).

### Undulation spectrum

The pushed membrane exhibits a different undulation spectrum from that of equilibrium. Figure 2b and c show the spectra of the tensionless membrane ($\gamma =0$) at $\Delta z_{\mathrm{fil}}=0.004$. At a low wave-number *q* (i.e., long wavelength), the membrane and filament surface connecting their tips have identical spectra. On the other hand, at a high *q* (i.e., short wavelength), the membrane spectra are not modified from the equilibrium spectrum in Fig. 2a, and the filament surface exhibits a flat spectrum of white noise, as in the absence of membrane–filament interactions. The threshold $q_{\mathrm{sep}}$ of separation of the spectra of the membrane and filament surface is correlated to the gap distance. With increasing $\Delta z_{\mathrm{fil}}$, $q_{\mathrm{sep}}$ increases, reaches a maximum when $\langle z_{\mathrm{gap}}\rangle$ is minimum, and then decreases (see Fig. S3). Up to the maximum of $q_{\mathrm{sep}}$, the filament spectrum at high *q* is flat. However, above the maximum, the filament spectrum is not flat at high *q*; thus, the spectrum is modified by the membrane–filament interactions (see Fig. S3c). These high *q* behaviors are not qualitatively changed by the choice of constraints (cases I or II). However, a distinct difference is found in the spectra of low *q*. In case I (tension constraint), the membrane undulation are suppressed at low *q*. In contrast, the spectrum is enhanced at $q \simeq 0.07\pi$ in case II (area constraint; compare Fig. 2b and c). Hence, the membrane buckles to maintain the membrane area *A*. A similar nonequilibrium buckling scenario resulting from the suppression of membrane fluctuations has been discussed as a mechanism that induces instability of the lamellar phase in shear flow^37^. Note that the membrane–filament interactions cannot be interpreted by the effective change of the surface tension, since the spectrum shapes in Fig. 2b and c are different from that for any value of $\gamma$.

**Figure 4:**
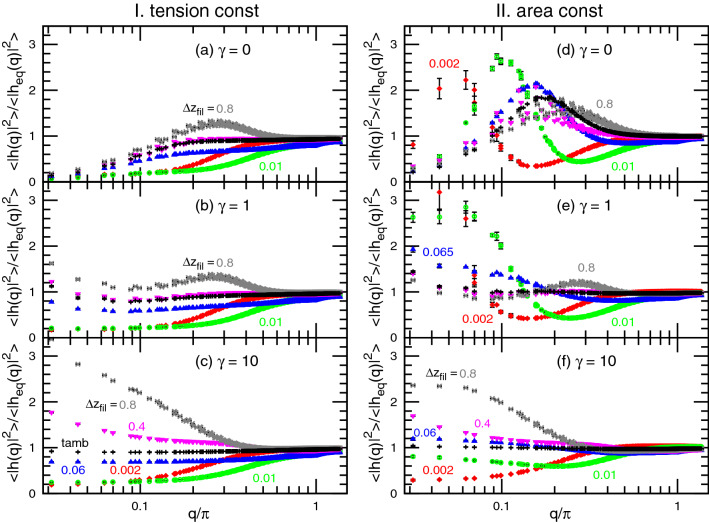
Undulation spectra normalized by the equilibrium values $\langle |h(q)|^2\rangle /\langle |h_{\mathrm{eq}}(q)|^2 \rangle$ for $\Delta z_{\mathrm{fil}}=0.002$, 0.01, 0.06 (or 0.065), 0.4, and 0.8. (**a**–**c**) Case I (tension constraint) and (**d**–**f**) case II (area constraint) for (**a**, **d**) $\gamma =0$; (**b**, **e**) $\gamma =1$; and (**c**, **f**) $\gamma =10$. The blue triangles represent the data at $\Delta z_{\mathrm{fil}}=0.06$ or 0.065, where the distance $\langle z_{\mathrm{gap}} \rangle$ has the minimum value. The black pluses represent the data of the membrane with the totally asymmetric diffusion (tamb). The error bars are smaller than the symbol size except for low wave-numbers at small $\Delta z_{\mathrm{fil}}$ in case II.

**Figure 5:**
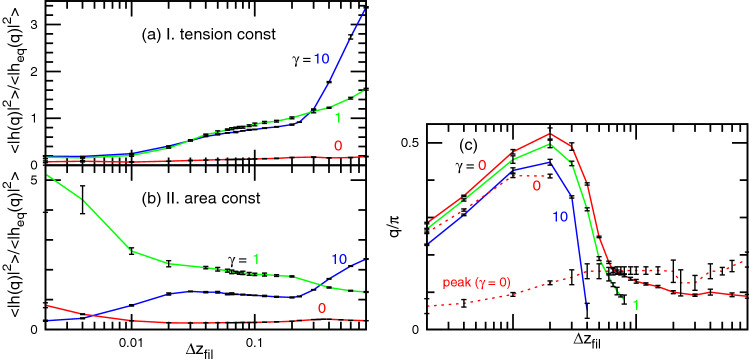
Dependence of the spectrum shape on ${\Delta z_{{{\text{fil}}}}}$. (**a**, **b**) Normalized undulation amplitude $\langle |h(q)|^2\rangle /\langle |h_{\mathrm{eq}}(q)|^2\rangle$ at the lowest wave-number $q=0.03125\pi$ for (**a**) case I (tension constraint) and (**b**) case II (area constraint). (**c**) The upper four lines represent the wave-number *q* at $\langle |h(q)|^2\rangle /\langle |h_{\mathrm{eq}}(q)|^2\rangle = 0.6$. The solid lines represent the data at $\gamma =0$, 1, and 10 in case I from top to bottom. The upper and lower dashed lines represent the data at $\gamma =0$ in case II for $\langle |h(q)|^2\rangle /\langle |h_{\mathrm{eq}}(q)|^2\rangle = 0.6$ and the peak of $\left\langle {|h(q)|^{2} } \right\rangle /\left\langle {|{h_{{{\text{eq}}}} } (q)|^{2} } \right\rangle$, respectively.

To further examine the spectrum changes, Fig. 4 shows the membrane spectrum normalized by the equilibrium spectrum as $\langle |h(q)|^2\rangle /\langle |h_{\mathrm{eq}}(q)|^2\rangle$. Let us first discuss case I, with tension constraint. For tensionless membranes low-*q* undulations are suppressed for all values of $\Delta z_{\mathrm{fil}}$, while it is slightly enhanced at the intermediate wave-number ($q \simeq 0.3\pi$) for large $\Delta z_{\mathrm{fil}}$ (Figs. 4a and 5a). A rough measure of the range of *q* for which the fluctuations are suppressed (i.e., $\langle |h(q)|^2\rangle /\langle |h_{\mathrm{eq}}(q)|^2\rangle <1$) during growth can be obtained from the evaluation of the value of *q* for which $\langle |h(q)|^2\rangle /\langle |h_{\mathrm{eq}}(q)|^2\rangle = 0.6$. This value of *q* plotted as the solid lines in Fig. 5c, is seen to exhibit a maximum for $\Delta z_{\mathrm{fil}}=0.02$. Hence, the low *q* range where the modes are suppressed is widest around $\Delta z_{\mathrm{fil}}=0.02$. This value is smaller than that corresponding to the minimum of the gap distance $\Delta z_{\mathrm{fil}}=0.065$. This maximum range of suppressed modes changes only slightly (see Fig. 5c) at higher $\gamma$. However, when $p_{\mathrm{fil}}$ is increased, the maximum of the suppression range increases while the corresponding value of $p_{\mathrm{fil}}\Delta z_{\mathrm{fil}}$ is constant (see Fig. S4). In addition to this suppression effect at small $\Delta z_{\mathrm{fil}}$, an enhancement effect is seen for large $\Delta z_{\mathrm{fil}}$ for high tensions. For $\gamma =10$ the undulation increases at $q \ll 1$ with increasing $\Delta z_{\mathrm{fil}}$ (see Figs. 4 and 5a), and a steep increase is obtained at $\Delta z_{\mathrm{fil}} \sim 1$

.

As a summary, for increasing $\Delta z_{\mathrm{fil}}$, there are first a range of modes that are suppressed. This range reaches a maximum for some value of $\Delta z_{\mathrm{fil}}$ (here $p_{\mathrm{fil}}\Delta z_{\mathrm{fil}}=0.01$), and then decreases. Upon a further increase in $\Delta z_{\mathrm{fil}}$, the low-*q* modes are enhanced for non-vanishing tensions.

Under the area constraint (case II), a similar trend of suppression of low *q* modes with a maximum at small $\Delta z_{\mathrm{fil}}$ together with an enhancement of low *q* modes at large $\Delta z_{\mathrm{fil}}$ is found. However, there is an important superimposed feature: a peak at low *q* appears in the spectrum ratio for small $\gamma$. The peak can be clearly seen in Fig. 4d for $\gamma =0$. The position of this peak, shown as the lower dashed line in Fig. 5c, shifts to a higher *q* with increasing $\Delta z_{\mathrm{fil}}$. For $\gamma =1$, the undulations are similar to those of $\gamma =10$ and $\gamma =0$ in cases I and II, respectively. Globally, the membrane undulation is strikingly changed by interactions with the filaments and strongly depends on the filament growth rate and membrane constraint types.

Moreover, we examined a totally asymmetric membrane (tamb) motion as a limited condition, in which the filaments are in complete contact with the membrane, and all of the downward membrane motion are rejected. The tamb spectra are close to those of the minimum gap distance, as shown in Fig. 4. Thus, the membrane under the minimum gap distance condition is well approximated by the asymmetric membrane motion. In this limit, the undulation due to the bending energy is modified ($\gamma =0$ and 1), while that due to the tension is not ($\gamma =10$). The tamb steady velocity $\langle v_z \rangle$ is $\simeq 50$% higher than that of the minimum gap distance condition: $\langle v_z \rangle = 0.0183$ (0.0190), 0.0180 (0.0182), and 0.0162 (0.0164) for $\gamma =0$, 1, and 10 in case I (case II), respectively. This difference is reasonable as a smaller minimum distance gives a higher velocity, as shown in Fig. S1.

### Vertical span

In the studies of surface growth, the time evolution and finite-size scaling of the surface thickness (or width) have been analyzed^1–6^. Here, we call this the surface vertical span to avoid confusion with the thickness of the membrane itself, and defined it as $z_{\mathrm{{span}}}^2=\sum _{i}^{N} (z_i-z_{\mathrm{G}})^2/N$, with $z_{\mathrm{G}}=\sum _{i}^{N} z_i/N$. Due to Parseval’s identity, it corresponds to the sum of the spectrum over all modes.

**Figure 6:**
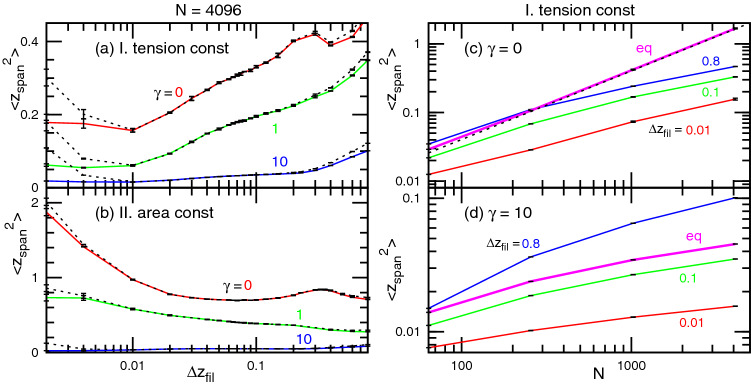
Vertical span $\langle z_{\mathrm{span}}^2 \rangle$ of the membrane and filament surfaces. (**a**, **b**) Dependence on $\Delta z_{\mathrm{fil}}$ for $\gamma =0$, 1, and 10 in (**a**) case I and (**b**) case II. The solid and dashed lines represent the data for the membrane and filaments, respectively. (**c**, **d**) Dependence on system size *N* for (**c**) $\gamma =0$ and (**d**) $\gamma =10$ in case I. The thick magenta lines in (**c**) and (**d**) represent the data at thermal equilibrium. The dashed black line in (**c**) indicates $\left\langle {z_{{{{\text{span}}}}^{2} }} \right\rangle = N/8\pi ^{3} \kappa$, which overlays the thick magenta line.

Figure 6 shows $\langle z_{\mathrm{{span}}}^2\rangle$ as a function of $\Delta z_{\mathrm{fil}}$ and the system size *N*. For very small or very large $\Delta z_{\mathrm{fil}}$, the filament vertical span is greater than the membrane span, reflecting the difference of the undulation spectra at high *q*. The $\Delta z_{\mathrm{fil}}$ dependence of $\langle z_{\mathrm{{span}}}^2\rangle$ is roughly captured by that of the undulation spectra at a low *q* (compare Figs. 5a, b and 6a, b). At a small $\Delta z_{\mathrm{fil}}$, $\langle z_{\mathrm{{span}}}^2\rangle$ provides slightly large and small values for $p_{\mathrm{fil}}=0.25$ and 1, respectively, while they merge well at large $\Delta z_{\mathrm{fil}}$ (see Fig. S1c and f). Under thermal equilibrium at $\gamma =0$, the membrane vertical span linearly increases as $\langle z_{\mathrm{{span}}}^2\rangle \sim N$ following the amplitude of the lowest undulation mode^30^. For the pushed membrane, this increase is reduced for all values of $\Delta z_{\mathrm{fil}}$ (see Fig. 6c). On the other hand, for $\gamma =10$, a greater increase is obtained at large $\Delta z_{\mathrm{fil}}$ (see Fig. 6d). This observation reflects an amplitude increase in the lowest mode as seen in Fig. 5a.

The excess membrane area increases as $\langle A\rangle /A_{xy}-1= (k_{\mathrm{B}}T/8\pi \kappa )\ln (N)+b$ for the tensionless membrane ($\gamma =0$) under thermal equilibrium^30,38,39^, where *b* is a constant. The modification of this size dependence by the filament growth is similar to that in the vertical span, and the excess area increase is largely reduced at $\gamma =0$ (see Fig. S5). Note that the mean velocity $\langle v_z\rangle$ and gap distance $\langle z_{\mathrm{gap}} \rangle$ exhibit negligibly small size dependence.

### Membrane dynamics

**Figure 7:**
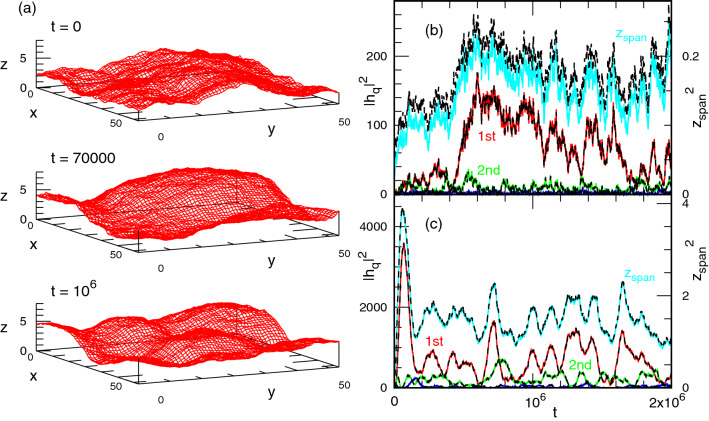
Initial time evolution of the membrane and filaments at $\gamma =0$ and $\Delta z_{\mathrm{fil}}=0.004$. (**a**) Sequential snapshots corresponding to the data in (**c**). (**b**, **c**) Time evolution of the amplitudes of the first, second, and third modes along the *x*-axis and the vertical span $z_{\mathrm{span}}$ in (**b**) case I and (**c**) case II. The solid and dashed lines represent the data of the membrane and filaments, respectively. The time unit is MC step. In both cases, a thermal equilibrium membrane conformation is used as an initial state; the initial filament surface is flat and contacts to the minimum position of the membrane.

Finally, we describe the time evolution. After the filaments contact the membrane, both surfaces relax into steady states. The choice of the initial conformations, such as a flat or thermal equilibrium membrane conformation does not lead to any notable difference in the subsequent dynamics. In most of the conditions, the undulations increase monotonously on average despite large fluctuations, as shown in Fig. 7b. However, a characteristic evolution is found at $\Delta z_{\mathrm{fil}}\lesssim 0.01$ for $\gamma =0$ under the area constraint (see Fig. 7a and c). First, the membrane and filament surface buckle together and form a bump, as shown in the middle snapshot in Fig. 7a. This bump leads to a strong peak in the first mode of the spectrum $|h(q)|^2$ and in $\langle z_{\mathrm{{span}}}^2\rangle$. Subsequently, the higher modes develop, leading to a steady state (the bottom snapshot in Fig. 7a). The initial suppression of the membrane undulation induces this overshoot buckling, because the lowest mode can evolve the fastest. It is known that a similar buckling at the lowest mode is induced by a negative surface tension^40^, so that it may be interpreted that an effective negative tension is yielded by the interaction with the filaments. Such buckling could share also similarities with wrinkles forming when a membrane is confined between two walls^41^, which are also be suppressed by tension^42^.

## Discussion

We numerically studied the membrane pushed by filament growth. The growth velocity has a maximum at a slightly larger filament-growth step $\Delta z_{\mathrm{fil}}$ than for the minimum gap distance; they are not sensitive to the surface tension and the constraint types. It is found that the membrane undulation spectrum is non-monotonously changed from that of thermal equilibrium. Under the tension constraint, the low-wave-number (long wavelength) undulations are suppressed for the tensionless membrane, and the range of the suppression displays a maximum at a value of $\Delta z_{\mathrm{fil}}$ that is smaller than that corresponding to the maximum velocity and minimum gap. At a high surface tension, this suppression is altered to enhancement with increasing $\Delta z_{\mathrm{fil}}$. Under the membrane-area constraint, we find similar features as in the tension constraint, but with two main differences for low tensions. First, a peak appears at some intermediate wave-number. Second, the membrane dynamics of relaxation to the steady-state is changed. The suppression of the undulation initially induces the buckling of the membrane at the lowest wave-number to maintain the membrane area. Subsequently, other modes are excited. The spectrum of the filament surface is identical to that of the membrane for low wave-numbers but deviates for high wave-numbers. The smaller the gap distance, the wider the identical region. Consequently, the vertical spans of the membrane and filaments deviate at small or large filament-growth steps.

Let us map the present model parameters to the membrane systems in living cells. In the model, $1/\ell _{\mathrm{fil}}^2$ is the lateral density of the filaments under the membrane. The membrane undulation is cut off at a membrane thickness of approximately 5 nm, so that the available range is $\ell _{\mathrm{fil}} \gtrsim 5$ nm. Our simulations clarified that the membrane undulation modes at high wave-numbers are not modified by the filament growth. Hence, we can conclude that the modes higher than $2\pi /\ell _{\mathrm{fil}}$ should also be unchanged, and we can only consider changes in the lower *q* modes.

An actin network pushes the plasma membrane of the leading edge in migrating cells^12,13^, in which the step size for actin growth is $\Delta z_{\mathrm{fil}}=2.7$ nm^17,18,25^. The density of actin filaments varies among cell types and with the cell state^12,13^. For high and low densities of $\ell _{\mathrm{fil}} = 10$ nm and 100 nm, $\Delta z_{\mathrm{fil}}/\ell _{\mathrm{fil}} = 0.27$ and 0.027 are obtained, respectively. Thus, at a high density, the discreteness of the filament growth may play a significant role in membrane–filament interactions. A microtubule is a hollow cylinder with a diameter of 25 nm, typically consisting of 13 protofilaments, and the growth unit length is $\Delta z_{\mathrm{fil}}=8$ nm^14,19^. When the microtubules are closely packed, the average distance is $\ell _{\mathrm{fil}} \simeq 7$ nm, such that $\Delta z_{\mathrm{fil}}/\ell _{\mathrm{fil}} \simeq 1$ at the maximum.

Here, we consider the minimal model for filament growth, in which the membrane and filament tips have only a repulsive interaction and no interactions between filaments. In the previous studies, the contact energy between neighboring filaments^20,21^, attractive potentials between the membrane and filament tips^22^, acceleration of filament growth by the membrane contact^27,28^, and filament rigidity^43^ have been investigated. These interactions can be easily added to the present model to clarify their effects on the undulations. Actin filaments often bind to membranes via curvature-inducing proteins^12^. Under such conditions, the filament contact is accompanied by the local induction of a membrane spontaneous curvature. Propagation waves can be generated by the coupling of the curvature-inducing proteins with the actin and/or regulatory proteins^44–46^. The effects of such a spontaneous curvature on membrane undulation are also an interesting topic for further studies. The model presented here could therefore be a versatile tool for the investigation of the interactions that may affect the membrane undulations in nonequilibrium conditions.

In previous studies^8–11^ on nonequilibrium membrane fluctuations, membrane undulations are always enhanced by active energy inputs. In the present case, the opposite effect (suppression) is also found. Indeed, filament growth can either increase or decrease the undulations depending on the conditions, and in particular, it can induce an excitation at a specific wave-number. Such undulations could stimulate characteristic length-scales or periodic structures that may give rise to filopodia and microspike coupling with the filament assembly.

## Methods

The fluid membrane is modeled by a squared mesh of *N* vertices with periodic boundary condition, as described in Ref.^33^. The bending energy is given by $U_{\mathrm{bend}} = \int (\kappa /2)(C_1+C_2)^2 dA$, where $C_1$ and $C_2$ represent the principal curvatures^29,47,48^. The Monge representation ($z = h(x,y)$) is employed, and the curvature is calculated as $C_1+C_2 = [(1+h_x^2) h_{yy} + (1+h_y^2) h_{xx} - 2h_x h_y h_{xy} ]/( 1+h_x^2+h_y^2 )^{3/2}$, where the subscripts represent spatial derivatives, such as $h_x = \partial h/\partial x$^29^. To control the membrane area, the intrinsic tension $\gamma _{\mathrm{it}}$ or the membrane area *A* is constrained by the addition of $U_{\mathrm{it}}$ or $U_{\mathrm{ar}}$ to the Hamiltonian in case I or II, respectively. To remove the artificial entropy production by the membrane tilt, a correction potential ${U}_{\mathrm{cor}} = - k_{\mathrm{B}}T \sum \ln (\cos \theta _{i})$ is also added to the Hamiltonian, where ${\theta_{i}}$ is the angle between the normal vector of the *i*-th site and the *z*-axis^33^. Straight filaments are arranged in the squared lattice ($x_i,y_i$) which is shared by the vertices of the membrane (see Fig. 1). Membrane–filament excluded-volume interactions are implemented by forbidding moves leading to inter-penetration ($z_{\mathrm{fil}}<z_{\mathrm{mb}}$).

In the filament growth step, one of the filaments is randomly selected, and its tip moves upward with a probability $p_{\mathrm{fil}}$ for a step of $\Delta z_{\mathrm{fil}}$. If the filament overlaps with the membrane vertex, the trial is rejected. The membrane vertex is moved by a vertical step taken from a uniform random number in $[-\Delta z_{\mathrm{mb}},\Delta z_{\mathrm{mb}}]$, and the motion is accepted or rejected by the Metropolis MC procedure. In each MC step, *N* trials are performed for both the filaments and membrane. In this study, $\Delta z_{\mathrm{mb}}=0.2$ and $K_A/A_0=1$ are used. For $\gamma =0$, 1, and 10, $\gamma _{\mathrm{it}}=0.44$, 1.44, and 10.4 ($A_0/N=1.036$, 1.022, and 1.01 for $N=4096$ in case II) are used, respectively. In our simulations, we have $p_{\mathrm{mb}}\approx 0.5$. More precisely, $p_{\mathrm{mb}}=0.5226$, 0.5128, and 0.4810 for $\gamma =0,1$, and 10 in case I and $p_{\mathrm{mb}}=0.5228$, 0.5130, and 0.4813 for $\gamma =0,1$, and 10 in case II, respectively. Error bars are calculated from three independent runs.

## Supplementary Information

Supplementary Information.

## Data Availability

The datasets generated during and/or analysed during the current study are available from the corresponding author on reasonable request.
