# Supplementary material for: Undulation of a moving fluid membrane pushed by filament growth
Source: Sci Rep. 2021 Apr 12;11:7985. doi: 10.1038/s41598-021-87073-6 (PMC8041810; doi:10.1038/s41598-021-87073-6)
Supplement: Supplementary file 1 — Supplementary Information. [file 41598_2021_87073_MOESM1_ESM.pdf]

Supplemental Material: Undulation of a moving fluid membrane pushed by filament growth

Hiroshi Noguchi[a,b*] and Olivier Pierre-Louis[b]

[a] *Institute for Solid State Physics, University of Tokyo, Kashiwa, Chiba 277-8581, Japan.* [b] *Institut Lumière Matière, UMR5306 Université Lyon 1-CNRS, Université de Lyon 69622, Villeurbanne, France.*

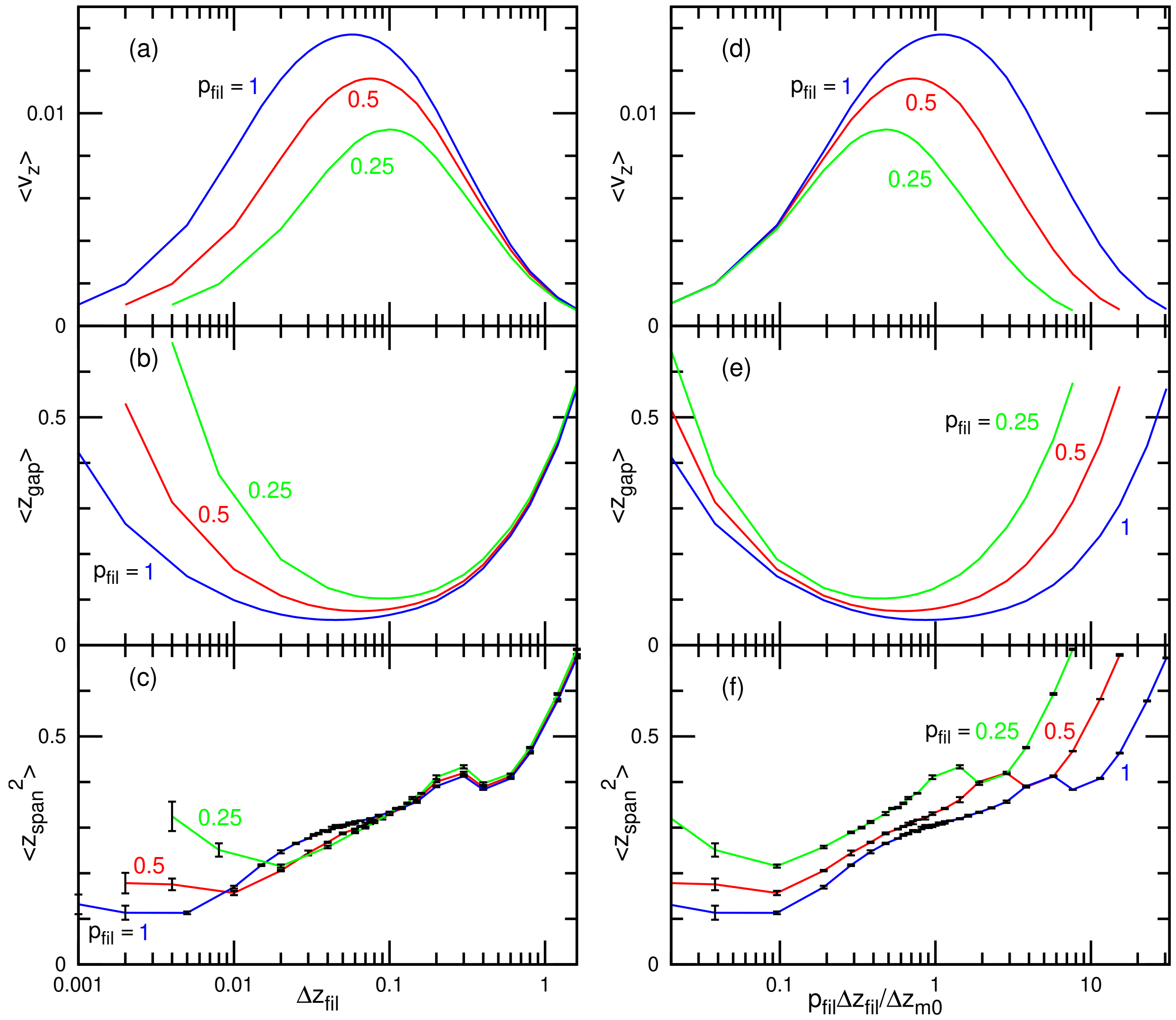

FIG. S1. Mean velocity $\langle v_z \rangle$, mean distance between the membrane and filament tips $\langle z_{\rm gap} \rangle$, and vertical membrane span $\langle z^2_{\rm span} \rangle$ as a function of $\Delta z_{\rm fil}$ for the growth probability $p_{\rm fil} = 0.25$, 0.5, and 1 at $\gamma = 0$ in case I (tension constraint). In (a)–(c), $\Delta z_{\rm fil}$ is used for the horizontal axis, while the mean filament growth distance $p_{\rm fil}\Delta z_{\rm fil}$ normalized by the mean membrane step $\Delta z_{\rm m0}$ is used in (d)–(f). The error bars in (a), (b), (d), and (e) are smaller than the line thickness.

* noguchi@issp.u-tokyo.ac.jp

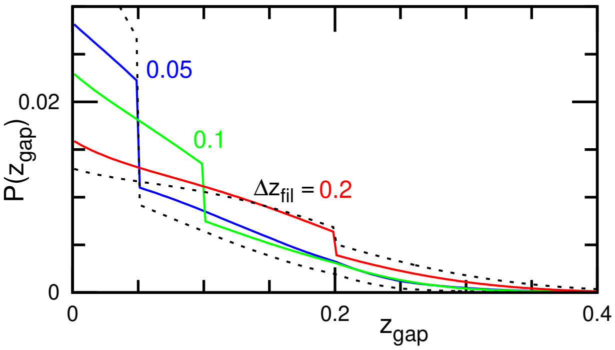

FIG. S2. Probability distribution of the distance between the membrane and filament tips, $z_{\rm gap}$, for $\Delta z_{\rm fil} = 0.05$, 0.1, and 0.2 at $\gamma = 0$ in case I (tension constraint). The solid lines represent the data for $p_{\rm fil} = 0.5$. The dashed lines represent the data for $p_{\rm fil} = 1$ at $\Delta z_{\rm fil} = 0.05$ and $p_{\rm fil} = 0.25$ at $\Delta z_{\rm fil} = 0.2$.

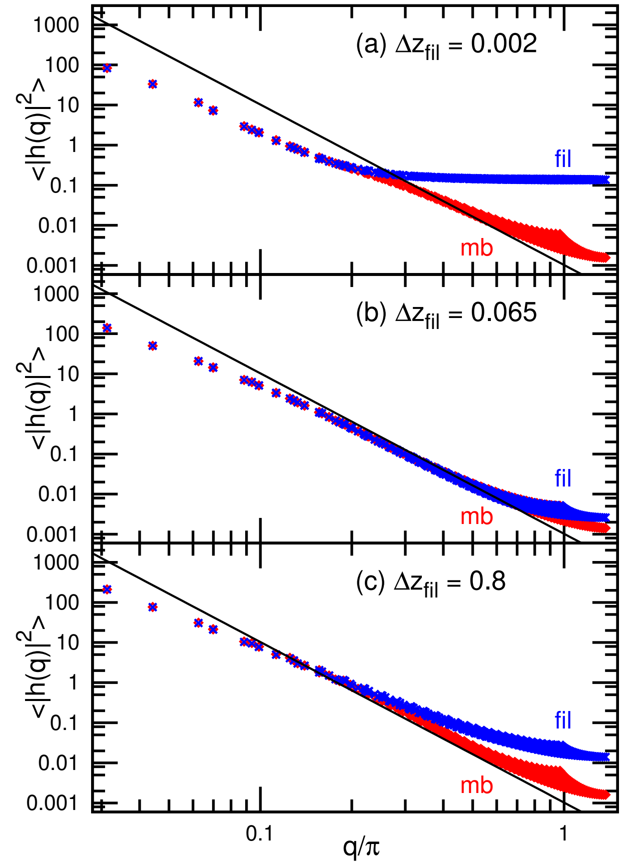

FIG. S3. Undulation spectra $\langle |h(q)|^2 \rangle$ of the membrane (red diamonds) and filament tips (blue cross marks) at $\gamma = 0$ in case I (tension constraint). (a) $\Delta z_{\rm fil} = 0.002$. (b) $\Delta z_{\rm fil} = 0.065$, where $\langle z_{\rm gap} \rangle$ has the minimum distance. (c) $\Delta z_{\rm fil} = 0.8$. Solid lines show $1/\kappa q^4$ with $\kappa = 10$. The error bars are smaller than the symbol size.

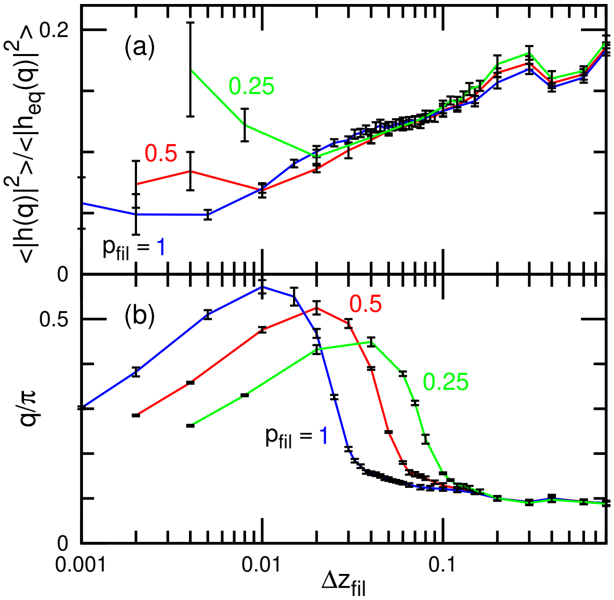

FIG. S4. Dependence of the spectrum shape on $\Delta z_{\rm fil}$ for $p_{\rm fil} = 0.25$, 0.5, and 1 at $\gamma = 0$ in case I (tension constraint). (a) Normalized undulation amplitude $\langle |h(q)|^2 \rangle / \langle |h_{\rm eq}(q)|^2 \rangle$ at the lowest wave-number $q = 0.03125\pi$. (b) Wave-number q at $\langle |h(q)|^2 \rangle / \langle |h_{\rm eq}(q)|^2 \rangle = 0.6$.

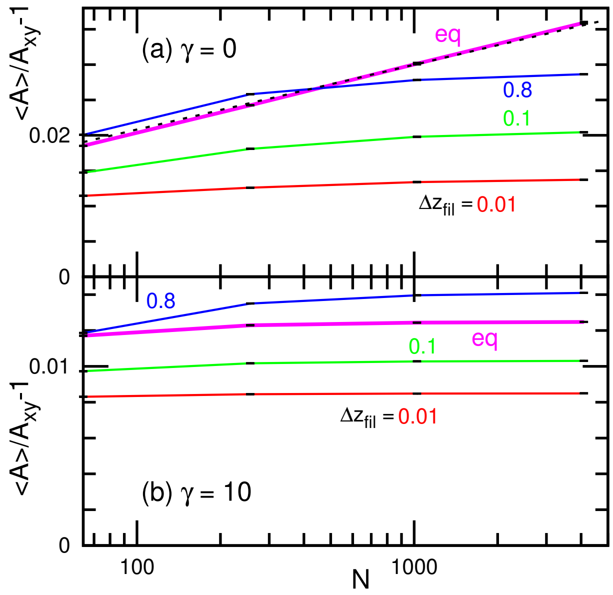

FIG. S5. Size dependence of the excess area ratio $\langle A \rangle / A_{xy} - 1$ at $\Delta z_{\rm fil} = 0.01$, 0.1, and 0.8 in case I (tension constraint). (a) $\gamma = 0$. (b) $\gamma = 10$. The thick magenta lines represent the data at thermal equilibrium. The dashed black line in (a) indicates $\langle A \rangle / A_{xy} - 1 = (1/8\pi\kappa)\ln(N) + 0.0025$, which overlays the thick magenta line.
